# Exploring adolescents’ and parents’ experiences of pharmacological and non-pharmacological treatment in ADHD: a qualitative study

**DOI:** 10.1186/s12888-026-08293-6

**Published:** 2026-06-13

**Authors:** Mobina Karimi, Sogand Ghasemzadeh, Peivand Ghasemzadeh, Keyvan Salehi, Faezeh Shabanali Fami

**Affiliations:** 1 Faculty of Psychology and Education, Department of Psychology and Education of Exceptional Children, University of Tehran, Tehran, Iran; 2 Department of Pharmacoeconomics and Pharmaceutical Management, TeMS.C., Islamic Azad University, Tehran, Iran; 3 Department of Curriculum Development and Instruction Methods, Faculty of Psychology and Education, University of Tehran, Tehran, Iran

**Keywords:** ADHD, Qualitative study, Phenomenological research, Adolescents, Parents, Pharmacological treatment, Non-pharmacological treatment, Treatment adherence, Lived experiences, Iran

## Abstract

**Background:**

Attention-Deficit/Hyperactivity Disorder (ADHD) presents major challenges for youth and families, including challenges related to treatment adherence and family experiences of pharmacological and non-pharmacological care. Additionally, while there is quantitative research on the efficacy of ADHD treatment, the qualitative aspects of lived experiences in youths treated, particularly in non-Western cultures, are less visible.

**Objective:**

This study sought to explore and understand the lived experiences of Iranian adolescents who have ADHD and their parents regarding pharmacological and non-pharmacological treatments with respect to barriers, facilitators, and conditions that affected the engagement.

**Methods:**

A descriptive phenomenological approach was used, based on Colaizzi’s seven-step method. Purposive criterion-based sampling was used to recruit nine adolescents (aged 12–16) diagnosed with ADHD and their parents (*n* = 10) from treatment centers in Tehran, Iran, and continued sampling until data saturation was reached. Semi-structured interviews (*n* = 19; average duration = 38.73 min) were conducted between June and August 2024, and interviews were transcribed verbatim and analyzed with MAXQDA 2024, generating 107 refined open codes from the interview data. Rigor was established by following Guba and Lincoln’s criteria for rigor, which included member checking and peer review.

**Results:**

From parents’ perspectives, two primary themes emerged: barriers and negative experiences (e.g., medication side effects, delays in diagnosis and treatment, and adherence challenges) and facilitators and positive experiences (e.g., increased ability to focus, trust in providers, and the role of non-pharmacological therapies). From the perspectives of adolescents, the key themes focused on the outcomes of treatment. These factors support or hinder adherence, non-pharmacological experiences, and therapeutic relationships, with considerations of benefits, such as increased concentration, and harms, such as stigma and fatigue. Cross-case comparisons identified similarities and differences in how parents and adolescents valued multi-modal approaches to ADHD treatment (e.g., medications and therapies), and the relative importance of autonomy vs. systemic support and connection with providers.

**Conclusion:**

The results highlight a clear need for person-centered, culturally sensitive approaches that account for the effects of medication side effects, stigma, and inaccessible treatment options to enhance adherence and quality of life. Implications include consideration for incorporating adolescent voices into shared decision-making and consideration of policy changes that allow access to comprehensive, subsidized ADHD care.

**Supplementary Information:**

The online version contains supplementary material available at 10.1186/s12888-026-08293-6.

## Introduction

Attention-Deficit/Hyperactivity Disorder (ADHD) is a common mental disorder diagnosed in childhood and adolescence [1]. ADHD is classified into three types according to the Diagnostic and Statistical Manual of Mental Disorders, Fifth Edition (DSM-5): inattentive type, hyperactive/impulsive type, and combined type. These types can be identified and diagnosed across different developmental stages [2]. In 2022, about 10.5% of children and adolescents (ages 3–17) had ADHD, and almost 60% of them had symptoms that were medium to severe. What’s more, about 75% of these people had at least one other mental health or comorbidities [3]. ADHD, particularly when it persists into adulthood, is associated with a reduced estimated life expectancy and adverse long-term outcomes in multiple life domains, including educational attainment, occupational functioning, health behaviors, and future planning [4].

In addition to being prevalent, ADHD has serious negative effects on individuals and society. It is associated with significant impairments across various life domains and makes people more likely to have mental disorders such as anxiety, depression, and substance use disorders. It can also cause academic, occupational, and social difficulties [5]. People with ADHD often experience poor academic achievement [6, 7], are less productive at work [7–9], and face serious challenges in social interactions, emotional regulation, and behavioral adaptation [7, 10–12]. During adolescence, the challenges of ADHD can intensify existing difficulties. This can lead to increased emotional distress and long-term behavioral consequences, including risks of social isolation, low self-esteem, and loneliness [6, 13–15]. When people with ADHD become adults, they are more likely to have poor mental and physical health, a lower quality of life, and increased risks of accidents, suicide, and premature mortality [5].

The impact of ADHD extends beyond the individual and deeply affects the family, particularly the parents. Parents who are raising a child with ADHD experience parental emotional exhaustion, disrupted parenting strategies, and heightened feelings of helplessness and psychological distress [16–19]. This is because the challenging behaviors associated with ADHD, such as difficulties with emotional regulation, sustained attention, and impulsivity, place demands on parents’ psychological and practical resources that exceed their available capacities, resulting in chronic strain, reduced self-efficacy, and parental burnout [20–22]. Multiple strategies have been proposed for managing ADHD. Usually, pharmacological treatment remains the first-line approach, followed by behavioral therapies, cognitive interventions, and neurofeedback [23–25]. Among these, stimulant medications, psychosocial therapies, or their combination offer the strongest evidence base [26–30].

Multimodal interventions for ADHD refer to integrative treatment approaches that combine pharmacotherapy with behavioral strategies—such as parent training, school-based support, and child-focused therapeutic techniques—to address symptoms across multiple contexts and promote broader functional improvements [31–33]. Pharmacological treatment is considered a cornerstone of multimodal interventions for ADHD, shown to effectively reduce symptoms, enhance daily functioning, and improve long-term outcomes [34–36]. These medicines have been linked to improvements in academic and occupational achievement, social functioning, behavioral regulation, and family harmony [37–40].

Some effective psychological interventions include Behavioral Parent Training (BPT), Behavioral Classroom Management, Organizational Skills Training, and Digital Therapeutics [41]. Neurofeedback, as a non-invasive approach, helps patients train their brain activity to enhance focus, attention, and self-regulation [42, 43]. There’s proof that certain polyunsaturated fatty acid supplements and micronutrients reduce ADHD symptoms in children and adolescents [44]. Dietary interventions, such as oligoantigenic, elimination, and additive-free diets, are also commonly recommended [45].

Despite proven effectiveness, medication adherence remains a major challenge [46]. Studies show that people across all age groups do not follow their ADHD medication regimens properly [47–49]. Various factors, including sociocultural and demographic elements such as stigma, lack of social support, and environmental context, influence this non-adherence [50, 51]. A big concern is about potential physical side effects such as appetite suppression, weight loss, and sleep disturbances [34, 35]. Also, clinical variables (e.g., severity of disorder, comorbidities), drug-related factors (e.g., tolerance, side effects), personal barriers (e.g., forgetfulness, altered sense of self), social constraints, limited access to mental health care, and the quality of interactions with healthcare professionals all play a role in treatment discontinuation [52–58]. The lived experiences of parents in raising a child with ADHD deeply influence their treatment decisions, often shaping their openness to non-pharmacological alternatives [59, 60]. Usually, these preferences stem from daily emotional strain, financial burden, social stigma, and a profound need for both emotional and practical support [59, 61, 62] Parents are showing more interest in different treatments like psychological therapies, neurofeedback, and nutritional interventions [63].

Despite the broad range of treatment options available, prior research has predominantly focused on quantitative measures, leaving the human and experiential aspects of ADHD—especially those of patients and their families—underexplored. Yet, a deeper understanding of these lived experiences is vital in identifying barriers and facilitators of treatment adherence, guiding the design of person-centered interventions, and supporting individualized treatment decisions. While previous qualitative studies have provided valuable insights into the experiences of either parents or adolescents with ADHD, they have typically focused on a single perspective or a single type of intervention [10, 64–67].

Our study goes beyond this by simultaneously and comprehensively exploring the lived experiences of both adolescents and their parents in relation to the full spectrum of treatment options—pharmacological and non-pharmacological—within a specific cultural context. This multidimensional and integrative approach, which has been largely absent from prior research, represents the key innovation of the present study. Therefore, the present study aims to explore and interpret the lived experiences of adolescents with ADHD and their parents, with a specific focus on the factors that influence their engagement with or withdrawal from pharmacological and non-pharmacological treatment options.

## Method

### Study design

This research is a fundamental qualitative study utilizing a descriptive phenomenological method. Descriptive phenomenology is applied when the researcher aims to comprehend and explain the meaning of a phenomenon or concept as lived by people in their everyday experiences [68–70]. It was opted for descriptive phenomenology since the main purpose of the study was to provide as accurate a depiction as possible of the treatment experience of adolescents with ADHD and their parents from their own perspective, not applying any additional interpretation. The choice of this methodology was due to its suitability when exploring the subjectivity, meaning, and perception associated with health, including the treatment process [71, 72]. In this study, adolescents with ADHD and their parents’ lived experiences are examined in detail, focusing on their perspectives on pharmacological and non-pharmacological treatments. The researcher aimed to interpret, explain, and accurately portray the participants’ accounts without adding personal inferences. Therefore, this research adopts a qualitative approach, employing descriptive phenomenology as its methodological framework.

### Study setting and participants

The research field of the present study encompasses all treatment centers in Tehran that provide services to individuals diagnosed with ADHD who reside in Tehran during the spring and summer of 2024. The inclusion criteria for this study were as follows: adolescent participants must be between 12 and 16 years old; they must have a formal diagnosis of ADHD confirmed by a licensed healthcare professional; they must have been undergoing pharmacological treatment for at least six months before the study; and their parents must have provided informed consent and demonstrated a willingness to share their experiences fully and accurately during the interviews. The exclusion criteria included: any adolescent or parent who wished to withdraw from the study at any point, or cases in which it was discovered during the research process that a participant no longer met the inclusion criteria (e.g., a change in diagnosis).

In this study, a purposive criterion-based sampling method was used to select several adolescents and their parents for participation, based on the inclusion criteria described above. In addition, maximum variation was sought across key characteristics, including adolescent gender and age, comorbidities, treatment history, and parental background, to capture a diverse range of experiences. The sampling process continued until thematic saturation had been reached. Data saturation was assessed separately for parent and adolescent interviews. After the final interviews in each group, no new codes or themes emerged, and the data repeated previously identified patterns. Because the study included both paired and non-paired parent–adolescent participants, parent and adolescent datasets were first analyzed separately and then compared to identify convergences and divergences.

In the analysis, it was observed that the concluding interviews echoed earlier remarks and offered no additional insights, indicating that data saturation had been reached after conducting interviews with nine adolescents and ten parents. Notably, for one adolescent participant (code IA-04), both parents were also interviewed: the mother (code IP-04) and the father (code IA-10). For the remaining participants, the mothers were interviewed, and their codes were aligned in parallel with those of their adolescent children. Table 1 presents the demographic characteristics of the participating parents, Table 2 outlines the demographics of the adolescent participants, and Table 3 summarizes relevant health and treatment-related information of the adolescent participants.

**Table 1:** Demographic characteristics of parents

Participant Code	Age	Gender	Education Level	Employment Status	Number of Children
IP-01	39	Female	High school diploma	Gym instructor	2
IP-02	43	Female	Master’s degree	Employee	2
IP-03	34	Female	High school diploma	Housewife	2
IP-04	44	Female	Undergraduate student	Student	2
IP-05	39	Female	Bachelor’s degree	Housewife	3
IP-06	36	Female	High school diploma	Housewife	1
IP-07	40	Female	9th grade	Self-employed	1
IP-08	39	Female	High school diploma	Housewife	2
IP-09	53	Female	High school diploma	Retired bank employee	2
IP-10	45	Male	Master’s degree	Company manager (remote work)	2

**Table 2:** Demographic characteristics of adolescents

Participant Code	Age	Gender	Grade Level	Family type	Birth Order
IA-01	13	Female	7th	Nuclear family	Twin
IA-02	14	Male	7th	Nuclear family	Second child
IA-03	12	Female	5th	Extended family	First child
IA-04	16	Male	9th	Nuclear family	First child
IA-05	14	Male	7th	Nuclear family	First child
IA-06	14	Male	7th	Nuclear family	Only child
IA-07	14	Male	7th	Nuclear family	Only child
IA-08	13	Female	5th	Nuclear family	Second child
IA-09	12	Male	5th	Nuclear family	Second child

**Table 3:** Information related to the health and treatment of adolescents

Participant Code	Age at ADHD Diagnosis (years)	Comorbid Disorders and Diseases	Daily Medication (*n*^1^)	Medication Dose (mg)	Medication Timing	Non-Pharmacological Treatments Received
IA-01	6	Stuttering, Anxiety	Lisdexamfetamine (2)	30	Morning and Evening	Play therapy, Occupational therapy, Speech therapy, Neurofeedback, Transcranial direct current stimulation (TDCS)
IA-02	13	Learning disorder	Lisdexamfetamine (1)	30	Morning	Educational psychotherapy
IA-03	11	Tic, Depression	Lisdexamfetamine (1)	50	Morning	-
			Sertraline (1)	50	Night	
			Pimozide (0.5)	4	Night	
IA-04	9	Oppositional defiant disorder (ODD), Sleep problems	sodium valproate (2)	200	Every 12 h	Neurofeedback, Behavioral therapy
			Sertraline (3)	50	Every 8 h	
			Tolterodine (2)	4	Every 12 h	
			Clonidine (1)	0.1	Night	
IA-05	9	-	Lisdexamfetamine (2)	30	Morning	-
			Risperidone (1)	1	Night	
IA-06	8	Anxiety, Depression	Methylphenidate Sandoz (1)	36	On a medication holiday	Neurofeedback
			Methylphenidate (1)	20		
			Atomoxetine (1)	25		
IA-07	6	History of seizures	Methylphenidate (1)	10	Discontinued due to seizures	-
			Risperidone (1)	1		
			Carbamazepine (2)	400	Morning and Night	
IA-08	7	-	Atomoxetine (1)	25	Night	Low Resolution Electromagnetic Tomography (LORETA), Cognitive training
			Donepezil (1)	5		
IA-09	9	-	Methylphenidate (1)	10	Morning	Neurofeedback

^1^Number of pills taken per day

### Data collection

Qualitative data were gathered through semi-structured individual interviews. Each session began with an explanation of the study’s purpose and assurances of confidentiality and participant safety. Interviews opened with broad questions about the participants’ lived experiences and gradually focused on their specific needs and challenges related to ADHD. Interview questions were designed to explore the outcomes of medication and factors influencing treatment adherence. Question development was informed by prior research and expert input from psychologists, psychiatrists, and pharmacists, who provided insights on medication safety, side effects, and usage [73].

The questions are given in Appendix 1 and Appendix 2. Data collection was conducted between June and August 2024 using participants’ preferred formats: face-to-face (4 interviews), telephone (9 interviews), and virtual (6 interviews via Google Meet, WhatsApp, Zoom, or video calls). These different modes may influence the level of rapport and depth of disclosure; however, efforts were made to maintain consistency by using the same semi-structured interview guide and ensuring a similar conversational approach across all formats. Interviews averaged 38.73 min. With consent, they were audio-recorded and transcribed into Word documents. A demographic questionnaire was also completed by parents, including: adolescent and parent age, adolescent gender, school grade, birth order, household composition, family medical/psychological history, socioeconomic status, parents’ marital status, education, employment, medication type, dosage, and schedule, and non-pharmacological treatments received.

### Data analysis

In this study, qualitative data were analyzed using Colaizzi’s seven-step method, a well-established strategy in descriptive phenomenological research [74–76]. The use of Colaizzi’s seven steps in this study is appropriate because the methodology allows for an in-depth understanding of the participants’ experiences with treatment, including their views regarding medications, non-medication treatment, and factors that influence treatment adherence. MAXQDA 2024 software was used for coding and organizing the data. The analytical process was conducted as follows:

1. **Familiarization**: The process began with immersion in the data through repeated reading of interview transcripts and listening to the audio recordings. Audio files were anonymized for ethical considerations. We carefully transcribed each interview verbatim, then imported the text into MAXQDA. We repeatedly reviewed both the transcriptions and recordings to ensure an in-depth understanding of the participants’ lived experiences.
2. **Extracting significant statements**: After reading all descriptions, we identified and highlighted phrases directly related to the phenomenon under study. These significant statements were marked as quotations within the software for further analysis.
3. **Formulating meanings**: We interpreted the significant statements by assigning meaning units (codes), while consciously setting aside our preconceptions to maintain the descriptive nature of the data. We then cross-checked each formulated meaning with its corresponding original statement to ensure accuracy and consistency.
4. **Clustering themes**: Redundant codes were eliminated, and related meanings were grouped into clusters. These clusters formed subthemes that reflected specific aspects of the participants’ experiences. The subthemes were then organized into overarching themes, representing distinct structural elements of the phenomenon (e.g., medication outcomes vs. school-related challenges).
5. **Developing an exhaustive description of the phenomenon**: The next step involved synthesizing the themes into a rich and detailed description that captured the essence of the participants’ lived experiences with the phenomenon.
6. **Producing the fundamental structure of the phenomenon**: The comprehensive description was distilled into a concise, essential structure. This representation included only the aspects considered critical to understanding the core of the phenomenon, expressed in the form of main themes.
7. **Validation**: To ensure the trustworthiness of the findings, we conducted a final validation step by returning to participants’ materials and consulting existing research in the field to confirm the accuracy and credibility of the extracted themes.

### Rigor/trustworthiness

To ascertain the validity of the qualitative data, Guba and Lincoln’s (1985) standards were utilized. These include credibility (internal validity), dependability (reliability), confirmability, and transferability (external validity) [77, 78]. To ensure maximum credibility, extended engagement with participants was maintained, and purposive sampling with maximum variation was used. Sampling continued until data saturation was achieved. Member checking involved providing interview transcripts and interview-derived codes to the participants for comment, and changes were made accordingly as required. This process was conducted with adolescent participants IA-03, IA-04, IA-09, and IA-08, as well as their corresponding parents (IP-03, IP-04, IP-10, IP-9, and IP-08). Any ambiguous information or misinterpretations were also resolved by follow-up telephone calls or SMS. In addition, qualitative research literature was being read, and field experts were being consulted to make sure that the interpretations were as close as possible to the participants’ actual lives. Training had also been conducted in two graduate-level phenomenological methodology workshops and qualitative data analysis software.

For dependability, all stages of the research process, including data collection, coding, and analysis decisions, were systematically documented to allow the research process to be traced and evaluated. To ensure confirmability, efforts were made to minimize researcher bias through critical reflection and the bracketing of personal assumptions during data collection and analysis. In addition, reflexive notes were maintained throughout the study to document researchers’ thoughts, assumptions, and analytic decisions. A comprehensive audit trail was established, including records of coding processes, theme development, and revisions during analysis. Furthermore, coding and theme development were conducted in close collaboration with multiple academic advisors, and interpretations were discussed and reviewed collectively to enhance analytical rigor and reduce the influence of individual bias. To aid in transferability, rich and descriptive accounts of the research setting, participant details, and main outcomes were provided so that readers could make informed decisions regarding applicability to other settings. Further, outcomes from the related literature on context were outlined to assist with an understanding of the context.

### Ethical considerations

Given the qualitative nature of the present research and its focus on rich analysis of the experiences of participants, compliance with ethical guidelines was given priority throughout the research process. To this end, ethical approval and a Clinical trial number were obtained from the Research Ethics Committee of the Faculty of Psychology and Education at the University of Tehran on 27 May 2024, under code IR.UT.PSYEDU.REC.1403.044. In addition, this research was conducted in accordance with the Declaration of Helsinki. Key principles respected included voluntary participation, informed consent, confidentiality, the right to withdraw, and special protection for adolescent participants as a vulnerable group. Participants were provided with a concise and straightforward explanation of the purposes, character, potential impact, and voluntary involvement in the study. Written informed consent was obtained from parents, and verbal assent was obtained from adolescent participants. The comfort and independence of adolescents were maintained throughout the interviews. Parents were not present during the adolescent interviews, and adolescents were not present during the parent interviews. Parents were also asked not to attend the adolescent interviews to ensure that adolescents could express their views freely and comfortably. Confidentiality and anonymity were assured, and identifying information was removed or anonymized to preserve participants’ anonymity. During the study, codes were used in place of real names, and the data were kept private in password-protected files that could be accessed only by research team members. Direct quotes were provided only with explicit consent and with caution not to reveal individual identities. Any potential distress at the mention of sensitive topics was valued, and participants were informed about their entitlement to access support services if needed. They were also told that they could withdraw from the study at any time without any consequences. Steps were taken to respect integrity, honesty, and transparency during data collection and reporting, devoid of falsification or falsity of data. The research participants were treated with dignity and could express their views openly without fear of being judged or retaliated against.

## Results

**Fig. 1:**
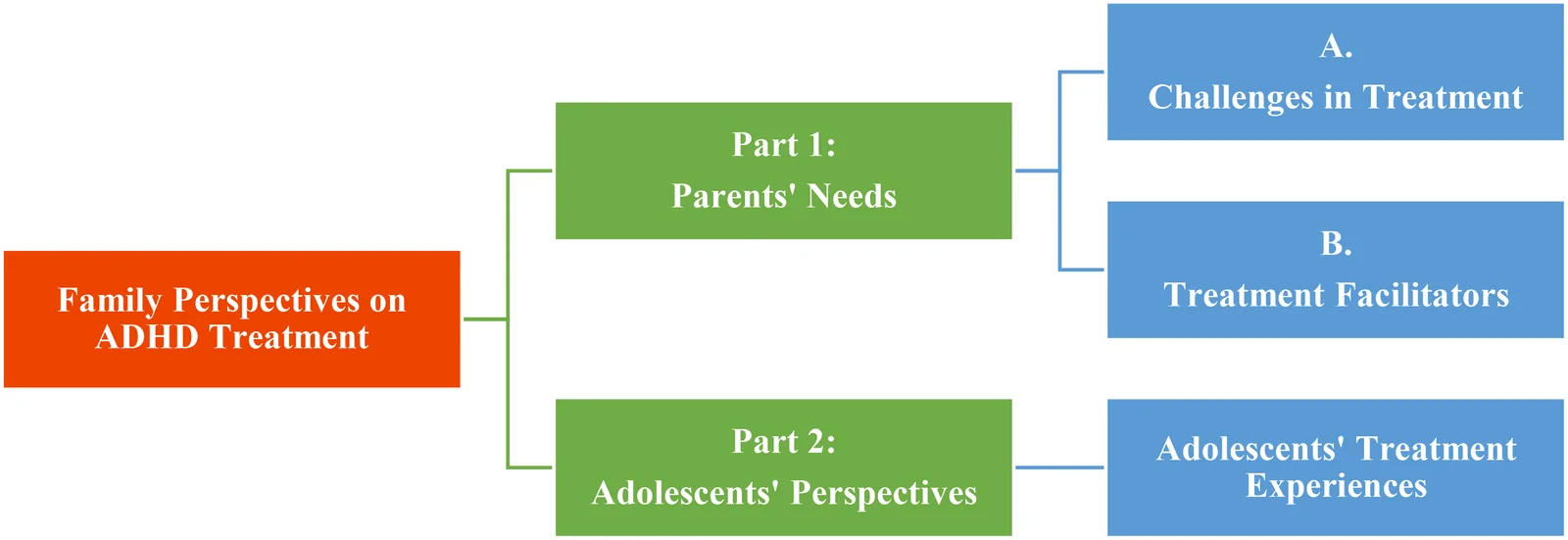
Family perspectives on ADHD treatment

The study findings are presented in two thematic sections, reflecting the perspectives of parents and adolescents, respectively, represented in Fig. 1. A word frequency analysis was conducted on the interview transcripts to gain a deeper insight into the findings. This method facilitated the identification of dominant patterns in the narratives of adolescents with ADHD and their parents, revealing recurrent concepts and terminology that underscore the core elements of their professional experiences and responsibilities. This qualitative study examined the experiences of adolescents with ADHD and their parents, identifying a total of **360 meaningful quotations**, which were further condensed into **572 preliminary codes**. After removing overlap and merging similar concepts, **107 refined open codes** remained, which were subsequently organized into subthemes and main themes. One main theme was identified from the adolescents’ data: **Adolescents’ Perspectives on Treatment** (n = 38, 35.5%). Two main themes were identified from the parents’ data, under the theme entitled **Parents ‘need**, that include **Challenges and Adverse Treatment Experiences** (n = 40, 37.4%), **Facilitators and Positive Experiences in Treatment** (n = 29, 27.1%).

### Part 1: parents’ needs

**Fig. 2:**
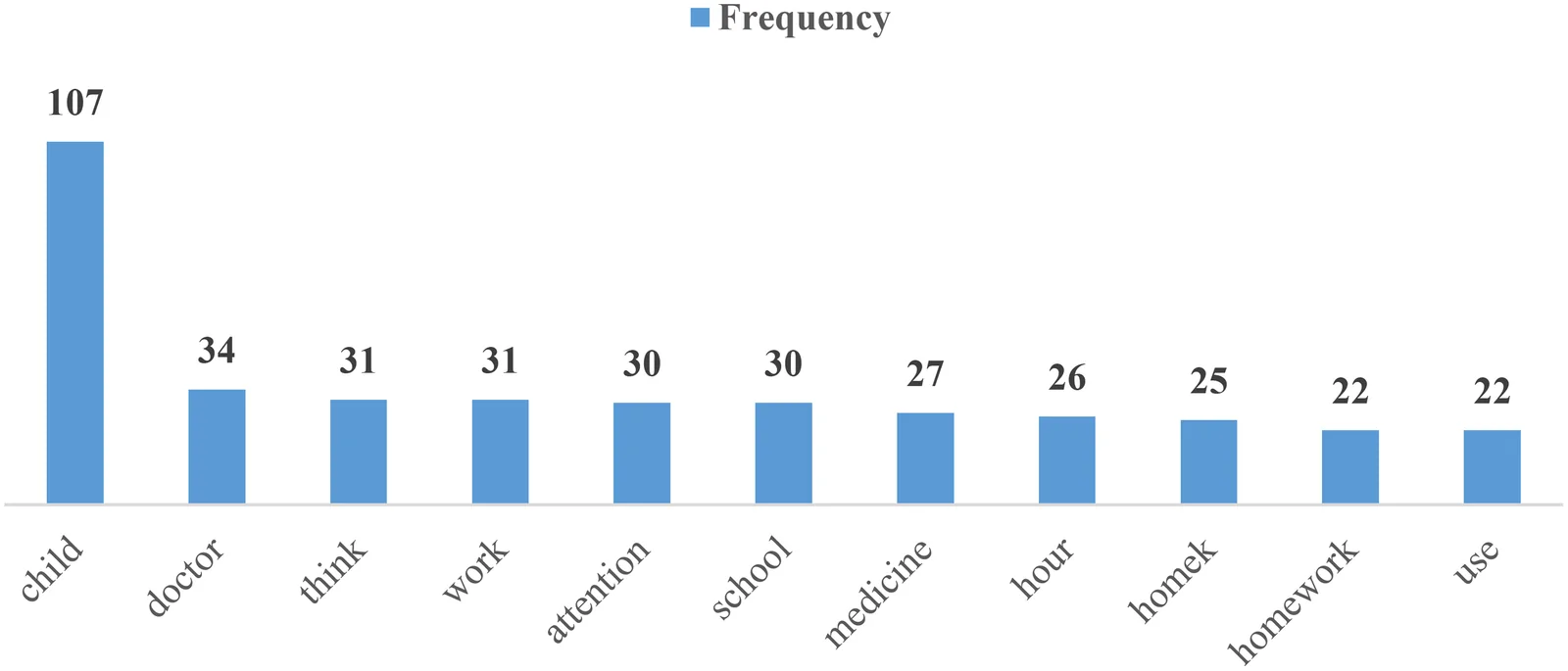
Frequency of parents’ interview words

A word frequency analysis of the parent interviews identified 505 unique words. Fig. 2 presents the most frequently used words in parents’ narratives. Analysis of word frequency in interviews with parents of teens having ADHD shows key themes shaping their experiences with treatment. The word “child” came up most often (107 times), showing that parents focused on their adolescents and the problems of handling their condition. The word “doctor” (34 times) shows that healthcare professionals are important in treatment, with frequent contact and reliance on them. Words like “think” and “work” (31 times each) suggest that parents are mentally and emotionally involved in solving problems and helping their child. Frequent mentions of “attention” and “school” (30 times each) stress worries about grades and focus, the main problems related to ADHD. The term “medicine” (27 times) stresses how important and complex drug treatment is in handling symptoms, with talks about how well it works and possible side effects. Also, words like “hour,” “home,” and “homework” show daily struggles with time, making a helpful home, and school duties. Lastly, the word “use” (22 times) probably points to using medication, plans, or other tools in treatment.

As a result of the data analysis, two main themes were identified from the parents’ data: Challenges and Adverse Treatment Experiences (sub-themes: Adverse Effects of Medication, Diagnostic and Treatment Challenges, Barriers to Treatment Adherence) and Facilitators and Positive Experiences in Treatment (sub-themes: Benefits of Pharmacological Treatment, Positive Diagnostic and Treatment Experiences, Enablers of Treatment Adherence, Role of Non-Pharmacological Interventions).

#### (A) Challenges and adverse treatment experiences (n^1^ = 40, 37.4%)

**Fig. 3:**
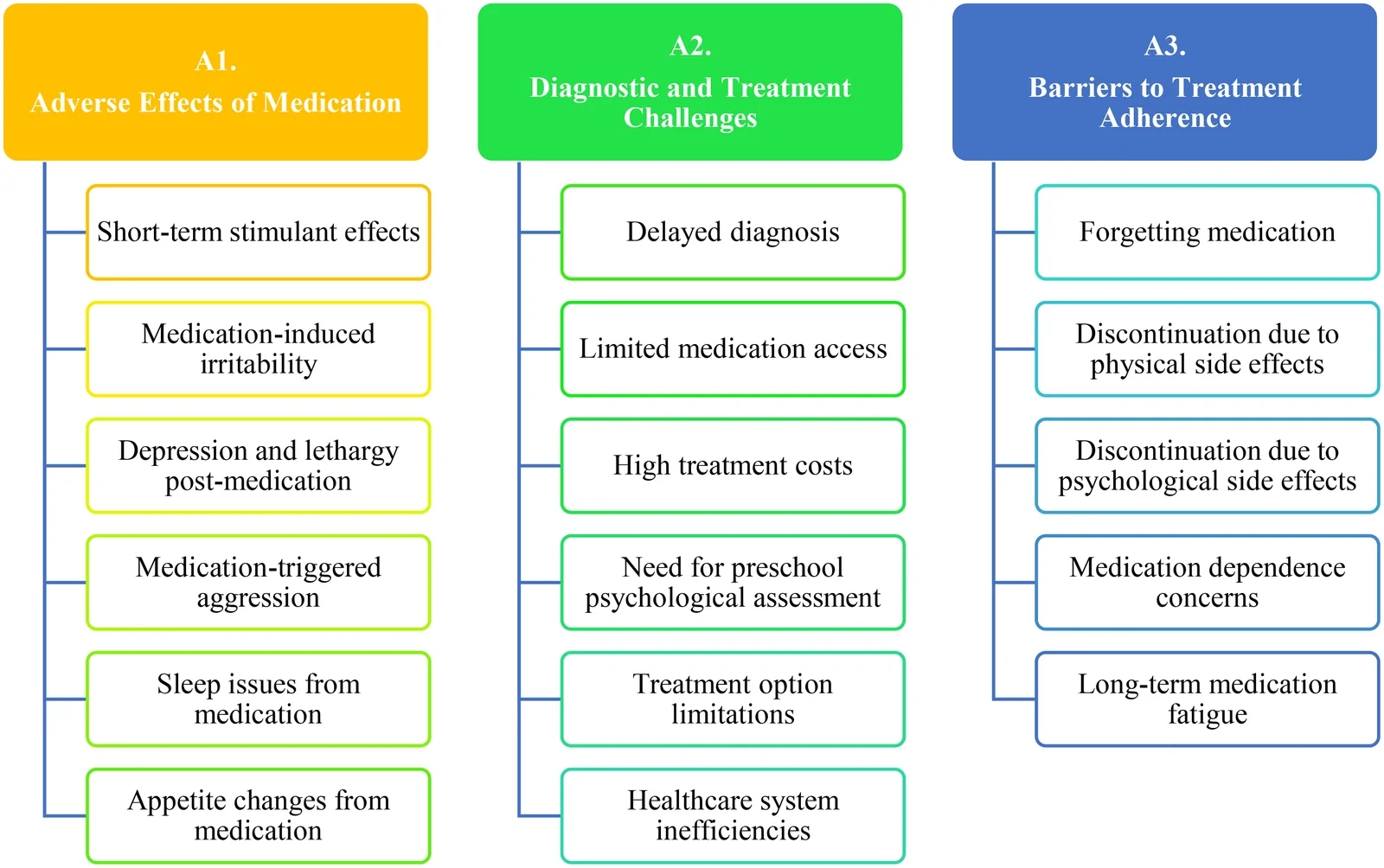
Parents’ needs: challenges in treatment

This theme covers parents’ perceptions of the challenges in diagnosing and treating their adolescents with ADHD. Parents described difficulty accessing proper care due to financial constraints, limited resources, and the inaccessibility of specialists or medication. Additionally, side effects—such as increased anxiety and mood issues—raised concerns and caused uncertainty and ambivalence about continuing treatment, often resulting in poor adherence. An overall overview of the theme: Parents’ Needs: Challenges in Treatment is summarized in Fig. 3.

##### (A1) Adverse effects of medication (*n* = 12, 11.21%)

Many parents described the limited and inconsistent effectiveness of stimulant medications such as Methylphenidate and Lisdexamfetamine, as well as several physical, psychological, and behavioral side effects. Parents indicated that children were more attentive for a brief period before they returned to distractibility, which disrupted schoolwork. Parents were concerned about the emergence of tics, changes in appetite, sleepiness, depression, withdrawal, and behavioral adaptation, like moodiness or hyperactivity. These adverse effects often resulted in reluctance to stay on the medicine. The statements below from participants encapsulate such experiences.

Ritalin was effective for about an hour or two… But as soon as the effect wore off, she would forget again that she had school, lessons, and homework. (IP-01)

When he took the pill, he wouldn’t say anything, he would just sit in a corner. I would say, ‘I wish I hadn’t given this pill to this child.’ (IP-04).

Sometimes he cried after taking the medicine and said he felt empty inside. (IP-06)

##### (A2) Diagnostic and treatment challenges (*n* = 14, 13.1%)

Delays in diagnosis and initiation of treatment for the children were discussed by parents during the interviews. Some attributed these delays to insufficient information or resistance to accepting the problems of their child in the beginning, which led to late intervention and worsening of the condition. Most parents reported that the diagnosis was made when the child began school, but they considered that earlier screening and identification, preferably before school entry, would have prevented subsequent academic and social issues.

It would’ve been much better if there had been some kind of screening before school. I think they should be diagnosed before first grade or at least have some kind of preparation. (IP-06)

We realized it too late. I think it’s because of this resistance. We were too late to believe that the child had a problem and that we needed to take action. If the first doctor had talked to us properly and given the right information, we would have taken action sooner. (IP-05)

The majority of the parents were not satisfied with the failure of therapy and counseling sessions to achieve improvement in the children. The failure annoyed the parents and, in some cases, resulted in the halt of treatment or the changing of treatment facilities. Parents believed therapists employed outdated or generic methods that were not tailored to meet their child’s specific needs. Additionally, the lofty costs of therapy sessions and the lack of adequate insurance coverage imposed a significant economic burden, which made it unfeasible to seek treatment regularly. These services were viewed as a waste of money and time by a few parents because their efforts were not yielding significant progress. Parents who had tried several interventions reported repeated disappointment and feelings of ineffectiveness. One of the fundamental issues, they believed, was that professionals lacked knowledge and expertise in addressing the unique needs of children and adolescents with ADHD. The parents, in their view, have to revamp the training system for psychologists and counselors so that practitioners would be better informed about understanding and dealing with the specific challenges of these children and adolescents.

Can the therapists prepare him for society?… It’s mostly theoretical, just academic talk. In my opinion, it’s all from textbooks, but in practice, there are no real results. Sure, psychology and medical books might give these issues a name, but there’s no real treatment. (IP-10)

In this part, parents have expressed complaints regarding the lack of proper support from government agencies for diagnosing and treating their adolescents with ADHD. They spoke of the shortage of quality, government-sponsored psychological services, the absence of free or low-fee specialist treatment, and the high cost of private consultation—all of which make it difficult for families to access useful services. Another concern among parents was that Iranian medicine was of inferior quality compared to foreign medicines. Even though they were aware of these limitations, families were often left with no other option but to use Iranian medicines due to the cost and unavailability of foreign medicines, leading to huge doubts about their efficacy and side effects.

I just feel like there should have been therapy, like a place supported by the government with proper facilities for treatment—somewhere they could tell us how to deal with him… Honestly, the costs were high. (IP-06)

##### (A3) Barriers to treatment adherence (*n* = 14, 13.1%)

This section of the findings talks about parents’ concerns and hurdles in the continuation of medication therapy for their adolescents with ADHD. The majority of parents feared the use of medicines in the long term since they observed some side effects, such as tics, mood changes, and physical aches and pains, or because they feared becoming addicted to or dependent on drugs. Such concerns have led some parents to look for alternative non-pharmacological interventions such as occupational therapy or play therapy.

Another key concern highlighted was forgetfulness or medication resistance, especially in older children. While the medicine is taken by younger children upon reminders from parents, adolescents tend to associate medication with a loss of control and refuse to take the same, resulting in fights within the home. The exorbitant financial burden of medication and limited access to quality foreign medicines also emerged as significant barriers, particularly for families who perceive domestic medications to be weaker. Parents, in some cases, associated frustration from decades-long administration of medication for many years, describing the experience as exhausting. Ultimately, certain parents questioned whether the benefits of medication truly exceeded its negatives. This has led various families to consider reducing or ending medication usage, especially when the treatment did not meet their expectations or negatively impacted the quality of their child’s life.

Sometimes I think these medications don’t help — instead, their side effects make her life even harder. I constantly ask myself if all this stress is worth it. (IP-03)

One of my friends is a lawyer and takes Vyas. She says it has a lot of side effects, and if the dosage goes above 30, it can become addictive. So, I’d prefer to gradually stop the Vyas and replace it with therapy or maybe play-based activities or something like that. (IP-02)

#### (B) Facilitators and positive treatment experiences (*n* = 29, 27.1%)

**Fig. 4:**
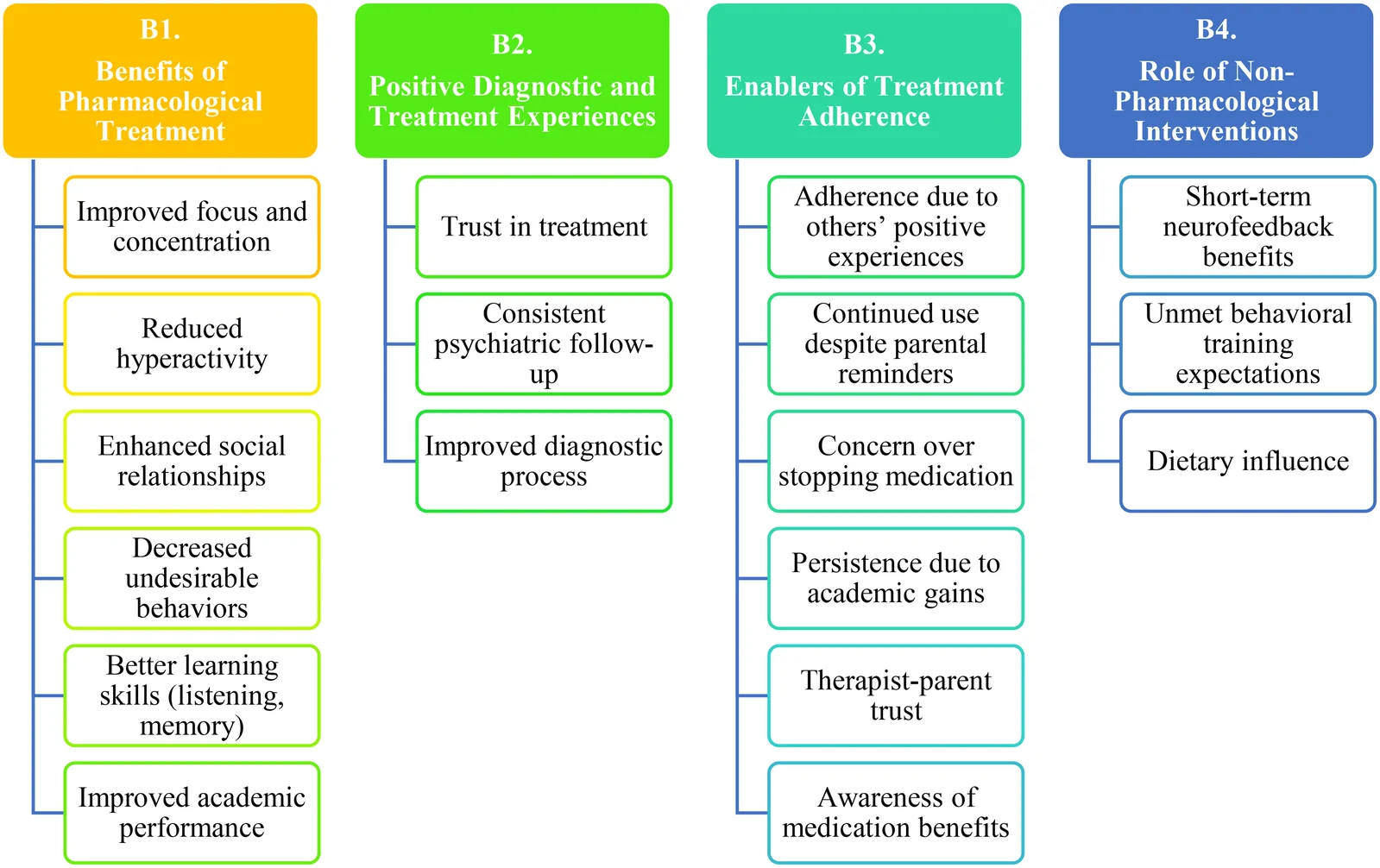
Parents’ needs: treatment facilitators

This section focuses on the progress made in the diagnosis and treatment of ADHD. The following discussion provides a detailed explanation of the factors that facilitate treatment adherence and highlights positive experiences in diagnosis and treatment, which not only help improve the adolescent’s condition but also give parents greater hope during the treatment process. Additionally, non-pharmacological strategies, such as behavioral management, complementary therapies, and cognitive training, are addressed, along with the beneficial effects of medication. The main categories of ‘Parents’ Needs: Treatment Facilitators’ are summarized in Fig. 4.

##### (B1) Benefits of pharmacological treatment (*n* = 10, 9.3%)

Once the treatment with medicine started, parents observed that children’s hyperactivity was declining. Teenagers also became much calmer and could handle things better in stimulating environments such as school and home. One of the most outstanding improvement areas observed was an enhancement of concentration and sustained attention toward school work, which led to improved academic performance, grades, and enhanced self-esteem. In addition, parents noticed that adolescents were more enthusiastic about homework and performed learning activities with more responsibility and interest. Parents also observed better listening and memory retention, with adolescents showing greater ability to follow instructions and recall learned material during class activities.

Medication also decreased aggressive and impulsive behaviors, such as yelling, hitting others, or sudden emotional reactions. These behavioral changes increased more harmonious family relationships and supported adolescents in being more constructively involved in social contexts. Reduced stubbornness and refusal to obey were also observed as an advantage. After medication was started, adolescents were more compliant and resisted fewer parent and teacher orders. Finally, a change was observed in the social behavior of the adolescents as described by their parents. The adolescents improved in communicating with other people, group participation, and performing more socialized behaviors. The improvements generated a higher sense of belonging, enhanced their self-esteem, and enabled them to acquire social skills that mattered most to them.

It makes others see him in a better light, and his communication improves… When he didn’t take his medication, he wouldn’t interact much with people. (IP-05)

They have some impulsive behaviors, like suddenly shouting or hitting someone while just passing by or playing. They don’t realize how intense the action or sound is. But with the medication, this had reduced.(IP-09).

The fact that he sat down and focused on his own — that was enough for me. He generally has difficulty with reading-based subjects, especially science. But this time, he studied by himself, and that was a huge change for me. (IP-02)

##### (B2) Positive diagnostic and treatment experiences (*n* = 5, 4.7%)

Parents universally pointed to the significance of diagnostic testing to accurately label the disorder of their children. They believe that proper diagnosis not only helps pinpoint the proper type of intervention but also gives parents a clear picture of what their child needs, thus enabling them to make sounder decisions. The second critical aspect of the treatment process was parents’ and medical professionals’ trust. Parents stated that when medical staff demonstrated commitment, follow-up, and empathy, they felt comfortable and assured that their children were properly cared for. Doctors who continually monitored medication and the child’s psyche and were good communicators with families were scored as being especially useful in convincing patients to continue treatment. Additionally, the clinical environment and atmosphere of the clinic were considered to be important. Parents indicated that a calm, supportive, and child-focused environment could reduce their child’s anxiety and promote improved cooperation with treatment. Struggling and persistent follow-up was also described as an important contributor to their positive results. Parents reported that even when initial treatments failed at first, ongoing visits to other specialists and exploring other options eventually meant change.

Trust is really important. We need to trust the psychologist, the psychiatrist, and the treatment itself so that we can apply it at home and see results. (IP-09)

Thank God, there’s been progress now in the diagnostic tests. There are both questionnaires and QEEG. I personally went and did all the tests, voluntarily. I even did neurofeedback. (IP-10)

##### (B3) Enablers of treatment adherence (*n* = 7, 6.5%)

Parents highlighted several important elements that enable treatment compliance in children and adolescents with ADHD. One of the strongest influences is how effective they perceive medication to be, with specific improvements in attention, learning, school work, social relationships, and fewer behavioral problems. Seeing these differences makes parents want to stick with the prescribed therapies. The second major factor is trust in health professionals, particularly among psychiatrists and psychologists. When parents believe in experts’ professionalism and dedication, they are more likely to cooperate with treatment and medication intake. Adverse experiences upon abrupt discontinuation of medicines, such as reinstatement of behavioral problems or heightened anxiety, have been sufficient to teach some parents the risks associated with suspension of treatment without medical supervision.

Several parents have also stated that children cannot manage their treatment by themselves, and therefore, active participation from parents is necessary. Parents are typically forced to be responsible for medication schedules, encourage compliance, and even use positive reinforcement strategies, like verbal reward, special activities, or extra family time, to maintain their children consistent with treatment. However, several parents mentioned that despite constant reminders and encouragement, children occasionally resisted taking their medication, requiring persistence and patience to maintain consistency. Education on the benefits and side effects of the treatment was also held to be essential. Parents who understood how the drugs act upon children were more resolute to commit to therapy. Informed decision-making helped them feel more in control and supportive of the treatment. Furthermore, hearing other families’ success stories about ADHD had a significant motivational effect. They provided hope and reinforced the value of long-term commitment to treatment. Finally, interaction with school staff—teachers, principals, and counselors—was highlighted as an essential support service.

When I trust my son’s doctor, I feel reassured that we’re on the right path. It makes it much easier for me to give him his medication and stay committed to his treatment because I know someone who knows what they’re doing is helping us. (IP-04)

Now they can at least understand things for a few hours on their own, but when they don’t take the medication, they don’t get anything — they can’t focus, they can’t sit still. (IP-01)

When I realized how much the medication was helping my son, I decided to learn more about it. We talked to the doctor about the side effects and benefits. Now I know that if he takes his medication on time, he can focus much better and do well in school (IP-07).

##### (B4) Role of non-pharmacological interventions (*n* = 7, 6.5%)

Some parents have used cognitive rehabilitation treatments like neurofeedback to reduce anxiety and improve focus, but many found the effects short-lived and the cost high. Behavioral therapies, such as occupational therapy and play therapy, have shown benefits when followed consistently over time. Additionally, some parents adjusted their child’s diet by reducing harmful foods like soda and sweets, and a few used herbal remedies like chamomile and orange blossom to help with anxiety and promote calmness.

The behavioral therapy sessions we’ve had so far didn’t work. The problem was that everything happened just in the clinic. They’d go in, something would be done, and then, once they left, it would be forgotten. Any action plan that was suggested would either be forgotten by the parents or resisted by the child. (IP-10)

I also started paying attention to his diet. For example, they said he shouldn’t drink soda, eat chocolate, or have chips and snacks at all. When he eats those, he becomes more hyperactive. (IP-07)

### Part 2: Adolescents’ perspectives on treatment (*n* = 38, 35.5%)

**Fig. 5:**
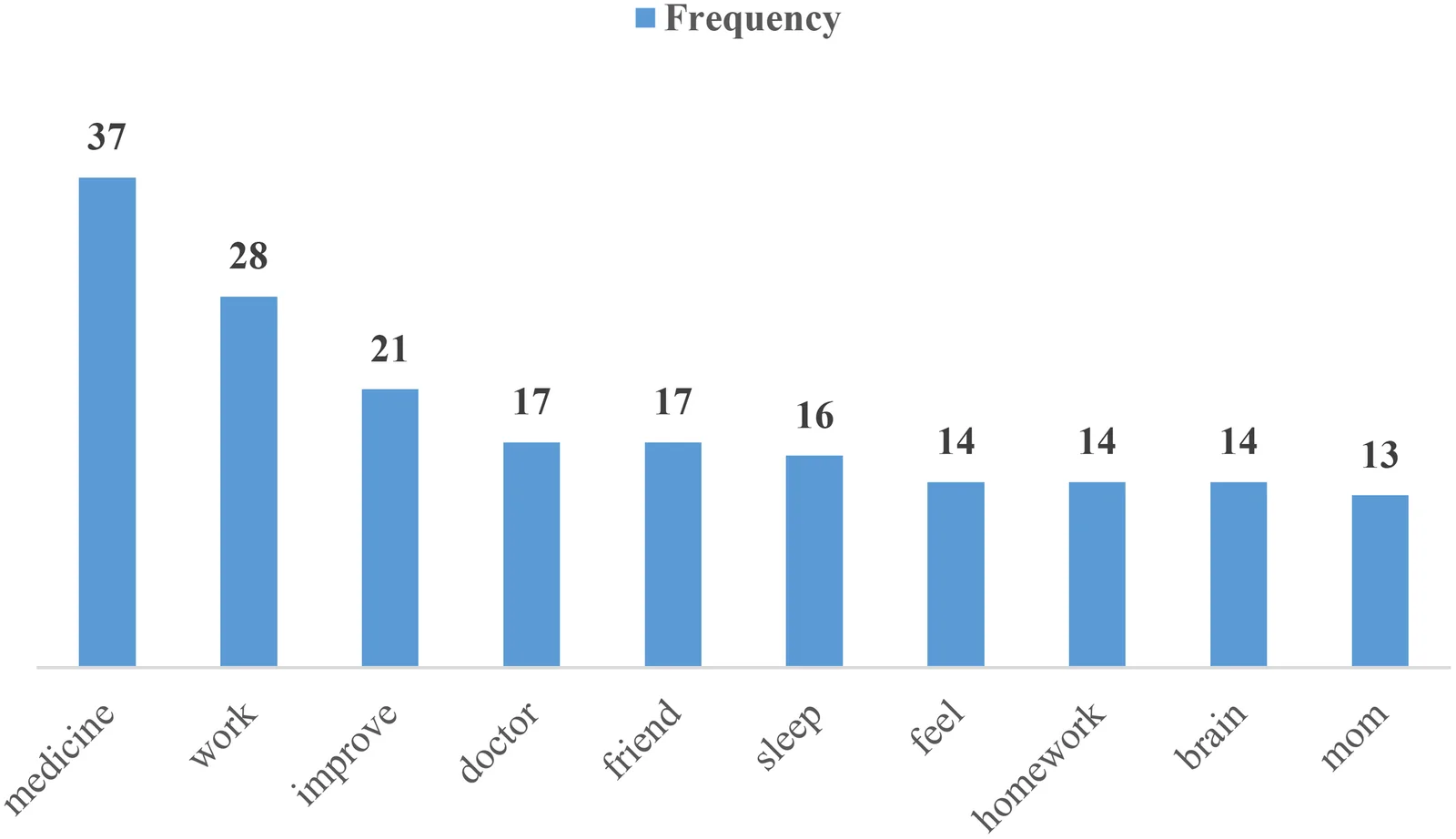
Frequency of adolescents’ interview words

Here, the perceptions of adolescents regarding treatment of ADHD are presented, including results of medication, predictors of compliance, and interactions with therapists and non-medication therapies. While most adolescents report that they are more focused and perform better at school with medication, side effects such as insomnia or loss of appetite may be challenging. Motivation is enhanced by trust and a sense of comfort in the therapeutic process, whereas negative experience lowers motivation. Behavior and cognitive therapy are generally perceived as beneficial, though perhaps not sufficient to manage symptoms. A word frequency analysis of the adolescent interviews identified 256 unique words. Figure 5 presents the most frequently used words in adolescents’ narratives.

**Fig. 6:**
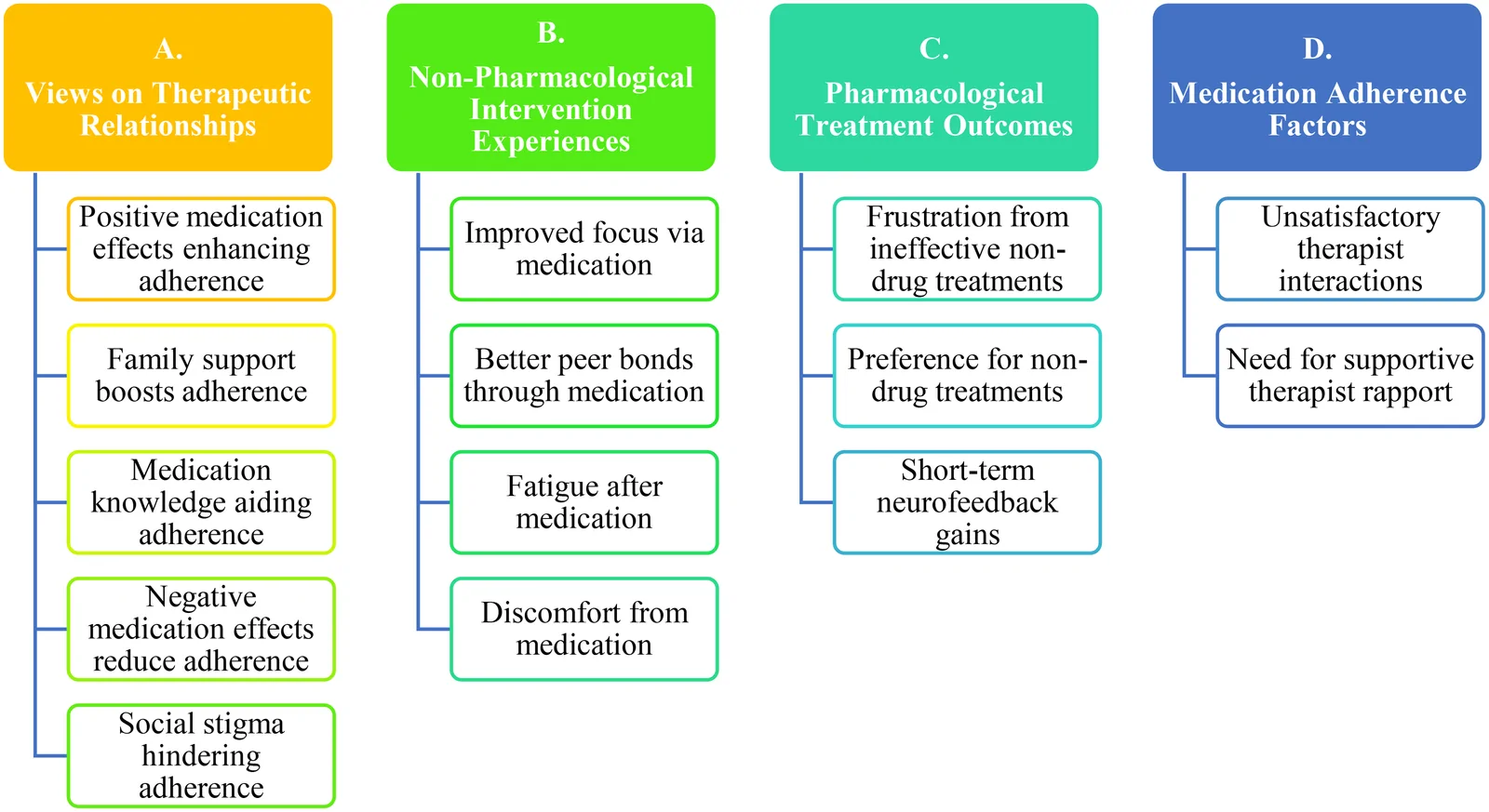
Adolescents’ perspectives: treatment experiences

Analysis of word frequency in adolescent interviews gives useful information on their experiences with ADHD and its treatment. The most common word was medicine (37 times), which shows how important medication is to them in handling their ADHD. This probably relates to the mental and physical results of taking medicine every day. The word work (28 times) probably means schoolwork, suggesting that school continues to be a major challenge. Likewise, improve (21 times) suggests a hopeful attitude, likely pointing to their wish to get better with their symptoms, grades, or general well-being. Words such as doctor (17 times) and friend (17 times) point out people important in their support systems: medical staff and friends. The mention of sleep (16 times) shows that sleep problems are a common issue for them, fitting with typical ADHD-related sleep problems. Other frequent words like feel, brain, and homework (14 times each) hint at a focus on how they are feeling, learning difficulties, and school responsibilities. The word mom (13 times) stresses how important parents are, especially mothers, in their daily support and treatment. As a result of the data analysis, one main theme was identified from the adolescents’ data: Adolescents’ Perspectives on Treatment (sub-themes: Views on Therapeutic Relationships, Pharmacological Treatment Outcomes, Non-Pharmacological Intervention Experiences, Medication Adherence Factors), displays in Fig. 6.

#### (A) Views on therapeutic relationships (*n* = 5, 4.7%)

Some adolescents reported negative experiences with mental health professionals, including being ignored, belittled, or even subjected to aggressive behavior. Such interactions led to feelings of loneliness, worthlessness, and mistrust toward the therapeutic process, ultimately reducing motivation to continue treatment. Adolescents expressed dissatisfaction when therapists failed to consider their individual needs and preferences, instead focusing solely on directives and parental perspectives. In contrast, those who developed a positive and supportive relationship with their counselor described feeling heard, respected, and safe. These adolescents highlighted the importance of collaborative and engaging sessions, such as talking, drawing, playing games, or working on concentration skills, which helped build trust and enhance emotional well-being. Positive therapeutic experiences contributed to increased self-confidence and encouraged adolescents to actively participate in their treatment.

I wish they would listen to me more. Sometimes parents just say things from their point of view, but if they actually listened to the kid, they’d do what the kid really wants. (IA-03)

Look, I say one thing, and the psychologist says something else. When I talk about my feelings, they just tell me to do this or that — it’s like they’re not even listening. (IA-06)

#### (B) Non-pharmacological intervention experiences (*n* = 5, 4.7%)

Adolescents shared various experiences with non-pharmacological treatments. Some expressed disappointment and frustration when previous therapies failed to produce visible change, which reduced their motivation and faith in future treatments. Overall, most preferred non-pharmacological options—such as counseling, play therapy, cognitive-behavioral therapy (CBT), and neurofeedback—over medication, mainly because these lacked side effects and allowed for emotional exploration in a more supportive environment. Neurofeedback was especially noted for producing short-term improvements in concentration and attention to schoolwork. However, some clients found the process either confusing or physically uncomfortable, which sometimes led to disengagement from these treatments.

The doctors we used to see didn’t really solve our problems. They just advised like ‘your house is messy’ or ‘you don’t listen to your mom,’ but nothing actually got better. (IA-01)

I think medications don’t always work and often have unpleasant effects. But when I work with my counselor or attend a class, I feel like they listen to me and help me get better. (IA-09)

#### (C) Pharmacological treatment outcomes (*n* = 16, 14.9%)

Several participants reported that medication positively affects cognitive functions, especially attention, concentration, and motivation for academics. Medication use was linked to improved cognitive abilities that led to greater academic engagement and productivity. Participants also noted improvements in behavior and increased ability to control emotions, resulting in better peer interactions and social functioning. However, medication treatment use was also accompanied by various physical and mental side effects. These included fatigue, drowsiness, appetite changes, dry mouth, thirst, insomnia, and in some cases, adverse reactions such as seizures. Psychological side effects like irritability or depression were also reported. Additionally, some adolescents experienced withdrawal symptoms like headaches and behavioral issues after stopping medication. The findings highlight that pharmacological treatment influences not only cognitive and academic performance but also emotional stability and physical health.

Since I started [the medication], I get angry much less often, and my classmates treat me much better now. I have a lot of friends these day (IA-08).

It just doesn’t make me feel good overall. Afterwards, I always feel drained and tired. (IA-04)

#### (D) Medication adherence factors (*n* = 12, 11.2%)

Several key factors facilitate adolescents’ adherence to pharmacological treatment for ADHD. Adolescents who experience a greater sense of control over their behavior and cognitive abilities after taking medication are more likely to adhere to treatment. The improved ability to concentrate and manage daily tasks strengthens their confidence and reinforces their commitment to the prescribed regimen. Second, family support, especially from parents, plays a critical role. Emotional encouragement, consistent reminders for medication intake, and practical involvement in managing side effects help adolescents remain engaged with their treatment. Third, adolescents with greater awareness of both the benefits and side effects of medications tend to experience less anxiety about using them and are more empowered in managing their condition. Access to clear, age-appropriate information about the purpose and effects of the medication supports a more informed and confident approach to treatment. Finally, a trusting and communicative relationship with healthcare providers significantly enhances adherence. When adolescents feel heard and respected, and when professionals take time to explain treatment plans and outcomes thoroughly, adolescents are more likely to view the treatment process positively and follow it consistently.

When I talked to him [doctor] and learned more about the medications, things got much better. They explained how the medicine could help me focus, make friends, do better in school, and make my parents happier. They also told me what would happen next. Now I feel better. (IA-01)

My family always supports me. When I take my medication, and my grades improve, they get really happy and encourage me to keep going. They always remember when it’s time for me to take my medicine, and when the side effects bother me, they talk to me and encourage me not to give up. (IA-06)

Why should I stop taking it when it helps me concentrate better? (IA-08)

Adolescents identified several factors that inhibit their medication adherence. Common side effects, such as fatigue, drowsiness, insomnia, appetite changes, and irritability, were often distressing and negatively affected their daily lives and social interactions. Some experienced more severe reactions, which caused fear and resistance to continuing use. Concerns about long-term dependency on medication and the belief that their cognitive functions only work with pharmaceutical help were also mentioned. Furthermore, the short duration of the medication’s effects discouraged consistent use, as some adolescents felt the benefits wore off quickly. Social stigma, especially in school settings, contributed to feelings of shame and isolation, leading some to skip doses to appear “normal” around peers. Finally, rigid treatment plans with little flexibility or regard for individual needs further decreased their motivation to adhere to the prescribed regimen.

Sometimes when I’m with my friends and talk about the medication, they keep asking questions like, ‘What is it for? What’s wrong with you?’ I feel like if I say I take medication, they’ll make fun of me or look at me differently. That’s why sometimes I prefer not to take it. (IA-01)

Sometimes the medication makes me lose my appetite, and I can’t sleep, or sometimes it makes me drowsy instead. Sometimes I think maybe I should stop taking it, but my parents don’t let me. (IA-04)

When I say I take medication at school between classes, some people give me weird looks, like I’m crazy. I feel embarrassed about taking medicine. I just want to be like everyone else and not have something in me that needs to be treated. (IA-07)

## Discussion

This qualitative research was conducted using a phenomenological approach to explore in depth the knowledge, perspectives, and concerns of families (including parents and adolescents) in Iran regarding pharmacological and non-pharmacological approaches to the treatment of ADHD. The study included 19 interviews with adolescents and their parents, and the analysis comprised 38,462 words and the identification of 107 refined open codes. open codes. The main focus of the study was on the views of parents and adolescents, examining key similarities and differences in the interrelationships among clinical treatments, personal experiences, cultural influences, and disruptive factors within the health care system.

The results of this study are presented in two parts: the first part focuses on parents’ needs, which are organized into two main categories—challenges and adverse treatment experiences including medication side effects, diagnostic and treatment challenges, and barriers to treatment adherence, and facilitators and positive treatment experiences including perceived benefits of pharmacological treatment, positive diagnostic and treatment experiences, enablers of treatment adherence, and the role of non-pharmacological interventions. The second part examines adolescents’ perspectives on treatment, including views on therapeutic relationships, outcomes of pharmacological treatment, experiences with non-pharmacological interventions, and factors influencing medication adherence.

This investigation aimed to provide deeper insights into a more effective understanding and monitoring of ADHD treatment. The findings illustrate how families select and implement treatment options for their children and how these decisions are shaped by cultural and social contexts, as well as factors that may interfere with medication adherence. It is important to note that while much of the existing literature emphasizes adult perspectives or reports provided by parents and teachers, this study foregrounds adolescents’ experiences and perspectives alongside those of their parents. This approach contributes to a more nuanced understanding of adolescents’ treatment-related needs and challenges, particularly in relation to medication adherence. This represents a methodological contribution, as it enables the identification and discussion of factors such as the perceived benefits and harms of medications, social concerns and stigma, and emotional and social supports that may optimize treatment adherence—an area of particular importance in ADHD research.

### Part 1: Parents’ needs

The findings of our study revealed that parents of adolescents with ADHD experience a wide range of challenges, including psychological distress, difficulties in treatment adherence, and concerns regarding medication use. Parents of children diagnosed with ADHD encounter psychological difficulties such as stress, anxiety, and lack of focus that can interfere with educational management programs, treatment compliance, and medication use [19, 79]. These findings are consistent with previous research indicating that parental psychological burden can negatively impact treatment processes and child outcomes. Parents of adolescents with ADHD could benefit from specific psychological services and educational program support to enhance parental mental state and cope with childcare difficulties [80]. In line with our results, improving access to such services may directly address the needs expressed by parents in our sample. Access to these services could improve factors such as parental sleep quality, leisure time, relationships, and mental health, leading to improved family quality of life [81].

In our study, one of the most prominent challenges reported by parents was related to the adverse effects and limited duration of effectiveness of stimulant medications. According to the results of both studies and clinical experience, stimulant medications that are prescribed to treat ADHD do have short-term side effects, including irritability, depression, lethargy, aggression, sleeping problems, and appetite changes. Stimulants are associated with other physical symptoms that overlap with some of these side effects, including fatigue, insomnia, headache, and dry mouth, and in severe instances, intensify restlessness and negative emotions. Furthermore, past studies confirm that stimulants are correlated with an array of side effects: dizziness, cardiovascular problems, psychosis, tics, and central nervous system (CNS) side effects [34, 82–84] and with types of “emotional” side effects, such as de-personalization or numbness, and decreased motivation as well [85]. These findings support the concerns expressed by parents in our interviews regarding mood changes, emotional blunting, and behavioral side effects. Therefore, to obtain better treatment outcomes, reducing or lessening complications and side effects would facilitate possible further work, suggesting the need for future research focused on optimizing medication regimens with fewer side effects. More experimentation in drug combinations, to attain drugs that are as effective but present a lower risk of complications and improve overall treatment quality.

Another key finding of our study was the presence of diagnostic delays, dissatisfaction with treatment services, and structural barriers such as high costs and limited access to specialized care. The body of literature evidences that families who have ADHD are at risk of delay in diagnosis, difficulties in obtaining quality medications without high costs, preschool psychological assessment, some treatment restrictions, and ineffective care. Delays in diagnosis arising from both an undervaluation and resistance from the parents’ level contribute to worsening academic and social problems in the teenager [21]. This aligns closely with our findings, where parents reported late recognition of symptoms and regret over missed opportunities for early intervention. Accessing a good quality medication only to use it during school time or difficulty tracking the efficacy of home remedies, however, with systemic and logistical barriers within a middle-income country, also adds to the family’s challenges [51]. Poorly planned and delivered counseling sessions, lack of close professional caseworker follow-up, poor attitudes of health care providers, and no case management coverage also served to intensify family dissatisfaction, fear, and anxiety, and challenged continuing the treatment itself [86]. Similarly, parents in our study described therapy as ineffective, overly theoretical, and not tailored to their child’s needs. Research demonstrates that families engaged in the management of ADHD contend with delays in the diagnosis and access to effective medications, the cost of health services, the requirement for a psychological assessment of preschoolers, limitations on treatment, and ineffective health services that are ineffective. Parents’ lack of awareness, followed by a denial to acknowledge their child’s issues, leads to a delay in diagnosis, which invariably results in academic and social problems during adolescence [21]. Our findings reinforce this pathway by illustrating how parental resistance and lack of early guidance contributed to delayed diagnosis. The family’s limited access to effective medications, the ineffectiveness of the available medications, systemic and logistical barriers, particularly in middle-income countries, all contribute to treatment challenges [51]. In effect, ineffective counseling sessions by professionals and a lack of continuing follow-up, along with negative attitudes by health care staff, increase the sense of fear or anxiety and dissatisfaction for families. The lack of insurance coverage worsens financial stress and deters the continuation of treatment [86]. Taken together, these findings highlight the need for systemic improvements, which was also strongly emphasized by parents in our study. These factors suggest there is a need to improve the health care infrastructure and educate the public.

In contrast to these challenges, our findings also identified several positive treatment experiences, particularly related to the benefits of pharmacological interventions. Meta-analytic studies provide compelling evidence for the efficacy of stimulant medications, including methylphenidate and lisdexamfetamine, regarding improvement of attentional focus and concentration, levels of hyperactivity, social relationships, and self-regulated and/or oppositional behaviors, and skills demonstrated within the educational context (such as listening and memory), and ultimately, academic success. These results are consistent with our findings, where parents reported improvements in attention, academic performance, and social functioning following medication use. In addition to symptom reduction, stimulant medications result in fewer aggressive behaviors, increased emotional self-regulation, and increased cooperation with parents and teachers [34, 87, 88]. Increases in concentration and improved memory translate into increased motivation and enjoyment about learning and completing homework, and also enhance family and social relationships, as evidenced in home and school environments [82, 89]. Similarly, parents in our study described improved family dynamics and greater engagement in school-related activities. Furthermore, a variety of consequential outcomes increases children’s levels of self-esteem, self-confidence, and emotional well-being, as well as their progress in and adherence to treatment, and overall quality of life [52]. Despite demonstrated success in treatment and support provided, additional individual variations in response to treatment call for investigating efforts for maximizing the effectiveness of medications [67].

Our study further highlighted the critical role of trust in healthcare professionals and continuous monitoring in facilitating treatment adherence. Parental trust in the therapeutic team, continuous monitoring by psychiatrists, and enhancement of assessment methods all positively impact adherence to treatment for ADHD, as demonstrated by studies. Trust in the treatment process instills feelings of hopefulness and encouragement to remain on treatment, especially in conjunction with parenting meetings that offer clear information regarding the benefits and harms of medications, and management of side effects [90]. This directly reflects our findings, where parents emphasized the importance of communication, empathy, and follow-up from clinicians. Meetings that addressed the needs of parents and provided education enhanced parents’ preparedness and confidence in continuing treatment [86, 91]. Education, daily reminders, behavioral therapies, and shared decision-making were also found to influence adherence positively [48].

In line with our findings on adherence barriers and facilitators, parents reported that both perceived effectiveness of medication and concerns about side effects influenced their decisions to continue or discontinue treatment. Research indicates that medication use resulting from the observation of medication with others, continued use due to parental prompts, concerns regarding the adverse effects of discontinuing medication, remaining on medication due to positive effects on academic performance, trust in the therapist and parents, and having an awareness of the benefits of medication, support compliance with treatment for ADHD. Emotional and practical support from parents, including encouragement to take medication and monitoring for adverse effects, increased an adolescent’s perceptions of security and motivation [86]. Consistent with our results, active parental involvement and reinforcement strategies were key facilitators of adherence. Trust in professionals, as well as clear communication about benefits and adverse effects, can alleviate concerns and provide an opportunity for informed decision-making [52]. Collaboration with schools and open conversation can also enhance adherence [61].

Our findings also highlighted the importance of school collaboration as a supportive factor. The enhancement of ADHD treatment adherence has beneficial implications for the quality of life of families. According to the research, the following factors increased adherence to ADHD treatment: taking the medication because of seeing the medication positively affecting someone else, remaining on treatment simply because of parental reminders, fear of the side effects of stopping treatment, staying in treatment because of positive effects on school work, having a trusting relationship with parents and the professional, and other factors that facilitate understanding that the treatment led to positive outcomes. In addition, the quality of parental emotional and practical support (e.g., monitoring for side effects, encouragement) improved adolescent feelings of safety and motivation [86]. Trusting the professional providing care and knowledge of the benefits and side effects associated with the medication helped alleviate anxiety related to making an informed decision [52]. Building relationships with their school and communicating with the school improved their adherence [61]. These aspects also positively impacted the quality of life of families. Despite measurable improvements in attention and behavior, maintaining long-term adherence remains challenging [92]. This challenge was also evident in our findings, where parents described ongoing struggles with sustained adherence over time.

### Part 2: Adolescents’ perspectives on treatment

The findings of our study indicated that adolescents with ADHD hold complex and sometimes ambivalent views toward treatment, shaped by both perceived benefits and negative experiences. According to research, an increase in positive medication outcomes (increased attention and performance), family support, and knowledge of the positive effects and side effects of medication can foster adherence to medication treatment for ADHD, whereas decreased adherence can occur in association with negative medication outcomes (negative side effects) and social stigma. These patterns were clearly reflected in our findings, where adolescents reported improved concentration and academic performance alongside concerns about side effects and peer perceptions. In another study, it was found that responses from parents can help facilitate adherence, such as the emotional and practical support of adolescents through reminder prompts to take their medication, thereby increasing adolescent motivation and feelings of safety around medication use [86]. Consistent with our results, parental involvement emerged as a central facilitator of adherence. The possibility of being aware of medication benefits and side effects through counseling also makes taking medication less difficult [52].

Our findings similarly showed that adolescents who had better knowledge about their medication felt more confident and less anxious about using it. On the other hand, side effects of medication and social stigma, particularly when associated with school settings, built momentum for resistance and non-adherence to medication [93]. This directly aligns with our findings, where adolescents described embarrassment, fear of judgment, and avoidance of medication use in peer contexts. Similarly, treatment plan inflexibility and lack of trust due to a non-belief, or skepticism about becoming dependent on the medication, are also barriers to medication adherence [94]. In our study, such concerns were reflected in adolescents’ resistance to rigid treatment plans and fears of long-term dependency. Research suggested that considering a non-stimulant medication, like an atomoxetine, as well as personalizing medication treatment, will facilitate adherence in adolescents with ADHD [95]. These findings highlight the importance of individualized treatment approaches, which was also implied in adolescents’ preference for flexible and tailored interventions in our study.

In addition to adherence-related factors, our findings demonstrated that adolescents perceive both cognitive and behavioral benefits from pharmacological treatment, despite experiencing notable side effects. The literature indicates that medication use in adolescents with ADHD leads to enhancement of attention span, positive and stable interactions with peers, but leads to feelings of fatigue and unpleasant affect (e.g., mood swings). Stimulant medication such as methylphenidate decreases hyperactivity, aggressiveness, and emotional dysregulation, which aids in academic performance and decreases family and social relationship tension [34, 82, 87]. These findings are consistent with our results, where adolescents reported improved focus, reduced impulsivity, and better social interactions. However, side effects such as insomnia, decreased appetite, and social stigma at school decrease compliance with treatment [88]. Similarly, adolescents in our study frequently reported sleep disturbances, appetite loss, and emotional discomfort as barriers to continued use. Alternatively, some teens may choose non-pharmacological treatments, such as neurofeedback, to avoid negative side effects, but the ineffectiveness and instability of the neurofeedback treatment diminish motivation [52]. This pattern was also observed in our findings, where adolescents preferred non-pharmacological approaches due to fewer side effects but reported frustration with their limited or short-term effectiveness.

Our findings further revealed that adolescents tend to have more positive attitudes toward non-pharmacological interventions, although these approaches are not always perceived as sufficient for symptom management. It has been shown in the literature that adolescents tend to drop out of treatment when they become frustrated with the instability of the non-pharmacological treatments, such as neurofeedback, and feel a sense of failure, though they have more positive feelings regarding the non-pharmacological treatments than the pharmacological treatments, and neurofeedback has shown a small positive short-term effect on concentration. This aligns closely with our findings, where adolescents expressed initial interest and comfort in therapies such as counseling and neurofeedback but reported reduced motivation when improvements were not sustained. Non-pharmacological treatments appeal to many parents because there are no side effects, and emotional and social skill-building is a central outcome of treatment exercises; however, participants often experience disappointment due to a lack of personalization and vagueness in the expected outcomes [41, 96]. Similarly, adolescents in our study criticized interventions that were not tailored to their individual needs or lacked clear outcomes. Specific to neurofeedback, participants may experience improved concentration; however, the sustainability of the treatment is limited [97]. Non-pharmacological treatments, such as CBT, building parent behavioral treatment (CBPT), and play therapy, focus on increasing emotional regulation, self-esteem, and social skills, but require continued practice to be effective [28, 98]. Mindfulness practices have been shown to decrease impulsivity [99]. Limitations surrounding access, cost, and social/cultural fit have proved to be barriers to effectiveness [100]. These barriers were also indirectly reflected in our findings through adolescents’ disengagement and inconsistent participation in such interventions.

Another important finding of our study was the critical role of therapeutic relationships in shaping adolescents’ engagement with treatment. Research indicates that adolescents’ feelings of annoyance towards their therapist and a perceived need for a close, friendly relationship with their therapist contribute to adherence to treatment for ADHD. Adolescents’ experience of positive, empathizing interaction, along with an environment where problems can be articulated without fear of judgment, increases self-esteem and quality of life, while situations of neglect and aggression create feelings of loneliness, fear, and stress, which are counterproductive to engaging in treatment behaviors [101, 102]. These findings strongly support our results, where negative therapeutic interactions led to mistrust and disengagement, while supportive and collaborative relationships enhanced motivation and participation. The support of parents or guardians by facilitating reminders, encouragement, and monitoring of symptoms and possible side effects enhances adolescents’ motivation and sense of security [46, 90]. Consistent with our findings, family support was a key factor in maintaining engagement with treatment. Counseling that explores the benefits and harms of medication can improve understanding and adherence to treatment [48]. While the positives of medications (such as improvement in academic performance and concentration) contribute positively to adherence, the negatives, which include side effects (such as fatigue and aggression) and social stigma at school, contribute negatively to adherence [52]. This dual effect of benefits and barriers was a central theme in our findings as well. Training of therapists can facilitate improved communication and reduce stigma through educational interventions [103]. Overall, our findings emphasize the need for adolescent-centered, empathetic, and flexible treatment approaches that address both psychological and social dimensions of ADHD management.

### Part 3: Comparative analysis of parents’ and adolescents’ perceptions of ADHD treatment

The findings of our study revealed both important convergences and notable divergences between parents’ and adolescents’ perceptions of ADHD treatment. Regarding the convergences and divergences in parents’ and adolescents’ perceptions of treatment for ADHD, this study discovered that pharmacological and non-pharmacological interventions both offered them benefits, but their perceptions regarding challenges and facilitators were quite contrasting indicating that treatment experiences are shared but interpreted differently across stakeholders.

Regarding the perceptions of pharmacological treatment, parents and adolescents both reported improvements in attention, control of behavior, and academic functioning after medication use suggesting a shared recognition of the effectiveness of pharmacological interventions. Parents, however, complained about side effects, dependence, and feelings of ineffectiveness of locally available medicines reflecting a more long-term, evaluative, and responsibility-driven perspective. Adolescents, despite reporting cognitive and social benefits, were most affected by physical and affective side effects such as insomnia, loss of appetite, and fatigue, which tended to decrease their adherence, highlighting a more immediate, experience-based evaluation of treatment. Social stigma and fear of labeling also influenced the degree of treatment adherence for adolescents, a factor that was largely absent in parents’ narratives but central in adolescents’ lived experiences.

Regarding factors that encourage treatment adherence, parents identified the role of trusting medical professionals, explicit behavioral change, psychoeducation, and cooperation with schools in upholding treatment, indicating a system-oriented and outcome-focused perspective. Adolescents, in contrast, emphasized autonomy, educationally informed treatment knowledge, family support, and professional respect as key factors reflecting a need for agency and involvement in decision-making processes. Both agreed that a relationship built on trust with providers significantly contributed to adherence, demonstrating a shared foundational mechanism despite differing expectations. Regarding experiences with non-pharmacological interventions, parents viewed behavioral therapies as potentially beneficial if implemented consistently, but frequently raised concerns about cost and limited effectiveness, suggesting a pragmatic evaluation based on resources and outcomes. Adolescents generally favored non-medication interventions because they were less invasive and allowed emotional expression, but reported frustration when interventions were not tailored to their needs or produced minimal results, indicating that perceived personalization is a critical factor for engagement in this group.

Regarding therapeutic relationships, while clinical competence and professionalism were central to parents, adolescents placed greater importance on empathy, active listening, and individualized care, highlighting a relational versus technical difference in expectations. Disempowering therapeutic relationships, such as feeling dismissed or unheard, discouraged adolescents’ engagement, whereas collaborative and interactive sessions enhanced trust and participation, suggesting that therapeutic alliance may function differently across developmental stages. Taken together, these findings suggest that although parents and adolescents share similar goals regarding symptom improvement, they differ substantially in how they evaluate treatment processes, prioritize outcomes, and engage with care.) (These divergences highlight the importance of adopting a family-centered and adolescent-sensitive approach in ADHD treatment, where both parental concerns and adolescents’ subjective experiences are actively integrated into treatment planning.) (Failing to address these differences may lead to misalignment in expectations, reduced adherence, and suboptimal treatment outcomes.

### Implications for clinical practice and policymaking

Building on the findings of our study, which highlighted differences between parents’ and adolescents’ perceptions as well as shared concerns regarding treatment effectiveness and side effects, our conclusions suggest that shared decision-making frameworks that incorporate adolescents’ perspectives are essential and should be increasingly adopted in clinical practice and policy, particularly given adolescents expressed need for autonomy, respect, and involvement in treatment decisions. For this reason, comprehensive psychoeducation should be prioritized and presented in an adapted from across cultural contexts to reduce misunderstanding and stigma associated with ADHD and its treatment. Consistent with our findings that both parental involvement and adolescent autonomy influence treatment adherence, involving the families continues to be important, although it is equally critical to recognize that adolescents have their own autonomy, and adherence must be considered a behavioral and communicative process rather than a purely medical one. Clinicians also must foster multimodal care tailored to adolescents’ individualized needs, as emphasized in our findings, and provide treatment, whether pharmacological approaches or non-pharmacological approaches, accordingly.

At the systemic level, our findings—particularly from parents—highlighted significant financial and structural barriers to accessing care. At the institutional level, financial hardships must be given immediate and serious consideration to advance the quality of services and improve access to effective treatments. Subsidizing ADHD medications, particularly high-quality medications, may alleviate inequities and address parental concerns regarding the effectiveness of locally available medications. Meaningful oversight of the domestic manufacturing system is needed to rebuild trust in the public system as lack of trust emerged as a key issue in our data.

Enhanced insurance coverage of non-pharmacological management options, including CBT and parent education, may serve to facilitate access to services and reduce reliance on medications which aligns with both parents’ and adolescents’ expressed interest in such interventions despite concerns about cost and effectiveness. Consistent with this position, educational systems, too, should be involved in establishing space for medication management within school settings, along with support programs to enhance academic performance, given that school-related challenges were central in both groups’ narratives; moreover, if the digital health strategies are culturally relevant and accessible, these strategies can assist individuals to adhere to treatment regimens.

Interestingly, our findings showed that participants did not spontaneously refer to digital health tools such as mobile applications, electronic reminders, or telehealth services, despite their potential relevance. While tools such as these can be useful, for example, in managing ADHD. Research related to other chronic conditions demonstrates that these types of tools may alleviate forgetfulness, an important discontinuity in the case of ADHD (Khan et al., 2024).

This gap may reflect infrastructural limitations, lack of awareness, or limited integration of such technologies within the local healthcare context. Although this may signal infrastructural inadequacy or models relevant to conditions and access in localized contexts in Iran, it does indicate a clear direction for both policy development and future research. It promotes the integration of advanced digital technologies to support mental well-being and disorder management in a more personalized and accessible manner. Ultimately, addressing both structural barriers and psychosocial factors, including stigma, may enhance the lives of individuals with ADHD and their families.

### Strengths, limitations, and future directions

The research reported here has strengths in its integration of parent and adolescent narratives providing a comparative and multi-perspective understanding of ADHD treatment experiences, and it utilized a rigorous phenomenological approach allowing for in-depth exploration of lived experiences. Limitations exist as well, particularly with its geographic focus on Tehran which may limit the generalizability of the findings to other cultural or healthcare contexts and the reliance on self-report, which might have been affected by recall bias or subjective interpretation of experiences. In addition, the qualitative nature of the study, while providing rich insights, limits the ability to establish causal relationships or generalize findings to larger populations. Future research should consider mixed and longitudinal designs to understand how treatment perceptions shift across developmental stages and treatment trajectories especially given the differences observed between parents and adolescents in this study. Quantitative validation will help in generalizing qualitative findings. Finally, it is important for research to address culturally appropriate non-pharmacological interventions and examine the role of digital tools to promote treatment adherence as both areas were either inconsistently experienced or underutilized according to our findings. In these ways, we can generate a better understanding and quality of treatments and develop more effective, patient-centered care models for ADHD.

## Conclusion

This research acknowledges the multidimensional and relational nature of treatment experiences of adolescents with ADHD and highlights that treatment experiences are situated in multiple influencing contexts. Each approach to treating ADHD has its own strengths; however, it is the fact that the pharmacological or non-pharmacological approach is available in practice that depends upon side effects, social labels, and financial factors. Parents’ concerns about medications and adolescents’ sensitivities to medication side effects—and therefore social factors—represent more than just a diverse reality; they suggest that both parents and adolescents with ADHD are asking for a more comprehensive or multidimensional approach to their treatment. They seek, on one hand, more accurate and transparent information about the potential benefits and harms of medications and alternative treatments, and on the other hand, they desire that treatments come with counseling and education that would help them appropriately respond to their children’s behavioral problems. To improve in the treatment of ADHD, care must be focused on individuals, quality, and a shared approach rather than strict guidelines or protocols, though these are helpful facets; cultural resiliency can only occur by trusting and collaborating with clients and their families. Given the complex nature of ADHD, clinicians and policymakers can initiate a more equitable, effective, and sustainable approach to health care for optimal ADHD outcomes, which improves the quality of life of a patient, an entire family, and a community.

## Supplementary Information

Below is the link to the electronic supplementary material.

Supplementary Material 1

## Data Availability

The datasets generated and analyzed during the current study are not publicly available due to the sensitive and identifiable nature of qualitative interview data and the need to protect participant confidentiality. However, anonymized data are available from the corresponding author on reasonable request and with permission of the Ethics Committee of the University of Tehran.

## Footnotes

1 n= indicates the total number of open codes.

## Abbreviations

ADHD: Attention-Deficit/Hyperactivity Disorder
DSM-5: Diagnostic and Statistical Manual of Mental Disorders, 5th Edition
BPT: Behavioral Parent Training
CBT: Cognitive-Behavioral Therapy
QEEG: Quantitative Electroencephalography
TDCS: Transcranial Direct Current Stimulation
LORETA: Low Resolution Electromagnetic Tomography
