# Supplementary material for: Exploring adolescents’ and parents’ experiences of pharmacological and non-pharmacological treatment in ADHD: a qualitative study
Source: BMC Psychiatry. 2026 Jun 13;26:656. doi: 10.1186/s12888-026-08293-6 (PMC13504824; doi:10.1186/s12888-026-08293-6)
Supplement: Supplementary file 1 [file 12888_2026_8293_MOESM1_ESM.docx]

1. **Appendix**

Appendix 1. *Interview Questions for Parents*

| *Interview Questions for Parents* | |
| --- | --- |
| 1 | When and with what signs did you start thinking about consulting a specialist? Who referred you to the specialist? |
| 2 | How would you describe your experience of the evaluation and diagnostic process? Which part was more difficult for you? |
| 3 | What medications has your child taken since the diagnosis, and what has been your experience with your child’s medication use? |
| 4 | At what times of the day does your child take medication, and what is your experience of its outcomes? |
| 5 | Usually, when does your child take medication, and how has this affected their tasks, schoolwork, or relationships? |
| 6 | What concerns do you have about your child taking medication? |
| 7 | What psychological treatments (e.g., psychotherapy sessions) has your child received, and what has been your experience with them? |
| 8 | What problems have you encountered in the course of treatment (whether medication or psychological) that you think, if improved, would make things easier for you and your child? |
| 9 | Among the treatments your child has received so far, which one do you consider more beneficial for them? Why? |
| 10 | How easy or difficult is it for you to follow up on treatment (such as taking your child to the doctor or ensuring medication use)? What makes you follow up more or less consistently? |
| 11 | How much of a role do you play in your child’s treatment, and what difficulties have you faced in this role? |
| 12 | How accessible are treatment services for you (in terms of cost, time, or distance)? Is it easy or difficult for you? |
| 13 | After the diagnosis, what kind of support did you receive from others or from the healthcare system? What new social problems have you faced? |
| 14 | What treatment needs does your child currently have that are not being met? |
| 15 | Finally, is there anything you would like to add, or any questions you would like to ask me? |

Appendix 2**.** *Interview Questions for Adolescents*

| *Interview Questions for Adolescents* | |
| --- | --- |
| 1 | When you first received the diagnosis, what things were difficult or important for you? |
| 2 | After the diagnosis, what kinds of treatments did you receive? |
| 3 | What was your experience with these treatments? |
| 4 | At what time of the day do you usually take your medication, and how does it affect your schoolwork, your friends, or your relationship with your family? |
| 5 | When you take medication, what good and bad effects do you feel? |
| 6 | What things in the treatment bother you that you think, if improved, would make treatment easier? |
| 7 | Among the treatments you have had so far, which one helped you more or made you feel more comfortable? Why? |
| 8 | Have you ever wanted to stop the treatment? If yes, why? What made you continue? |
| 9 | When you visit a psychologist or psychiatrist, how do you feel? What has your experience been like? |
| 10 | How do your teachers and peers, or friends, treat you when they know you are under treatment? |
| 11 | How do your parents, brothers, or sisters feel or behave when they know you are under treatment? |
| 12 | Is there anything else you would like to say that I have not asked? Or is there something you would like to ask me? |
